# Very Late-Onset Post-transplant Lymphoproliferative Disorder Presenting as Small Bowel Obstruction After Initial Remission in a Living-Donor Kidney Transplant Recipient

**DOI:** 10.7759/cureus.106873

**Published:** 2026-04-12

**Authors:** Yu Shimada, Daiji Takamoto, Yuri Hasegawa, Takeaki Noguchi, Takashi Kawahara, Jun-ichi Teranishi

**Affiliations:** 1 Department of Urology, Yokohama City University Medical Center, Yokohama, JPN

**Keywords:** diffuse large b-cell lymphoma, kidney transplantation, post-transplant lymphoproliferative disorder, small bowel obstruction, very late-onset

## Abstract

Post-transplant lymphoproliferative disorder (PTLD) is a lymphoid malignancy that develops in the setting of immunosuppression after solid organ transplantation. Although the incidence of PTLD after kidney transplantation is relatively low, late-onset cases are increasingly recognized with improved long-term graft survival. Very late-onset PTLD, occurring more than 10 years after transplantation, is often Epstein-Barr virus (EBV)-negative and frequently presents as monomorphic disease such as diffuse large B-cell lymphoma (DLBCL).

We report a case of very late-onset EBV-negative monomorphic PTLD occurring 19 years after ABO-compatible living-donor kidney transplantation. A 49-year-old woman with stable graft function was found to have generalized lymphadenopathy on routine imaging. Lymph node biopsy confirmed CD20-positive DLBCL. After reduction of immunosuppression, she achieved complete remission with rituximab monotherapy. However, two months later, she developed small bowel obstruction due to a newly emerged intestinal mass, and histopathology confirmed recurrent DLBCL. Despite surgical resection, disease progression was observed with a mesenteric lesion. She subsequently received cyclophosphamide, doxorubicin, vincristine, and prednisone (CHOP) followed by rituximab + CHOP (R-CHOP) chemotherapy, achieving complete remission while maintaining stable allograft function.

This case highlights that very late-onset PTLD may behave similarly to de novo DLBCL in immunocompetent patients. Early relapse after rituximab monotherapy should prompt consideration of lymphoma-standard chemotherapy, particularly in EBV-negative monomorphic PTLD, while carefully balancing graft preservation.

## Introduction

Post-transplant lymphoproliferative disorder (PTLD) is a lymphoid neoplasm arising in the setting of immunosuppression after solid organ transplantation. The incidence of PTLD after kidney transplantation is estimated to be approximately 1-3% [1]. PTLD is broadly classified into early-onset and late-onset disease, which differ in pathogenesis, histological features, and prognosis. Early-onset PTLD is strongly associated with Epstein-Barr virus (EBV) infection due to impaired EBV-specific immune surveillance under immunosuppressive therapy [2]. In contrast, late-onset PTLD, particularly cases occurring more than 10 years after transplantation, more frequently present as monomorphic disease such as diffuse large B-cell lymphoma (DLBCL) and are more often EBV-negative [3]. Approximately 50-60% of late-onset cases are EBV-related [4]. However, the precise mechanisms underlying late-onset PTLD remain unclear.

Management of PTLD generally begins with reduction of immunosuppression followed by rituximab-based therapy, although the optimal treatment strategy remains controversial, particularly in EBV-negative monomorphic disease.

With improvements in transplant outcomes and long-term graft survival, the number of recipients surviving more than 10 years after transplantation has increased, leading to growing recognition of very late-onset PTLD. We report a case of very late-onset EBV-negative monomorphic PTLD occurring 19 years after living-donor kidney transplantation, which recurred as small bowel obstruction shortly after initial remission.

## Case presentation

A 49-year-old woman with a history of spina bifida had performed clean intermittent catheterization since childhood. She underwent ABO-compatible living-donor kidney transplantation from her elder sister at the age of 29 due to chronic kidney disease secondary to reflux nephropathy. Induction immunosuppressive therapy consisted of basiliximab, tacrolimus, mycophenolate mofetil, and methylprednisolone.

Her post-transplant course was uneventful with stable graft function. Because of a desire for pregnancy, mycophenolate mofetil was replaced with azathioprine. Although pregnancy was not achieved, maintenance immunosuppression with tacrolimus (6.4 mg/day) and azathioprine (50 mg/day) was continued due to stable renal function.

**Figure 1:**
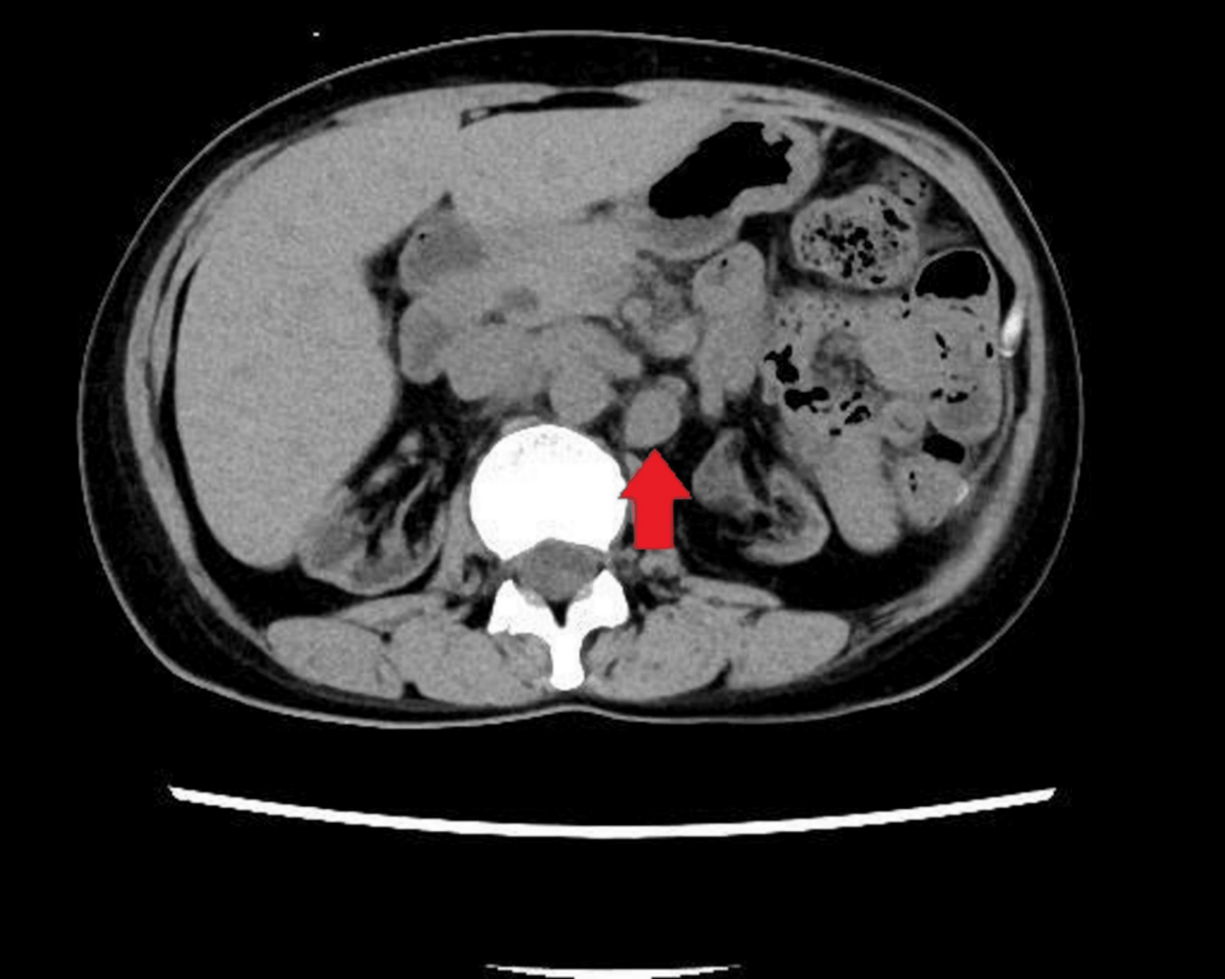
Contrast-Enhanced CT Before Rituximab Therapy Contrast-enhanced computed tomography performed at the time of referral to our institution before initiation of rituximab therapy demonstrates intra-abdominal lymphadenopathy (arrow), suggestive of lymphoproliferative disease.

Approximately 19 years after transplantation, routine contrast-enhanced computed tomography (CT) revealed intra-abdominal lymphadenopathy (Figure 1). The patient was asymptomatic at the time of initial diagnosis.

**Figure 2:**
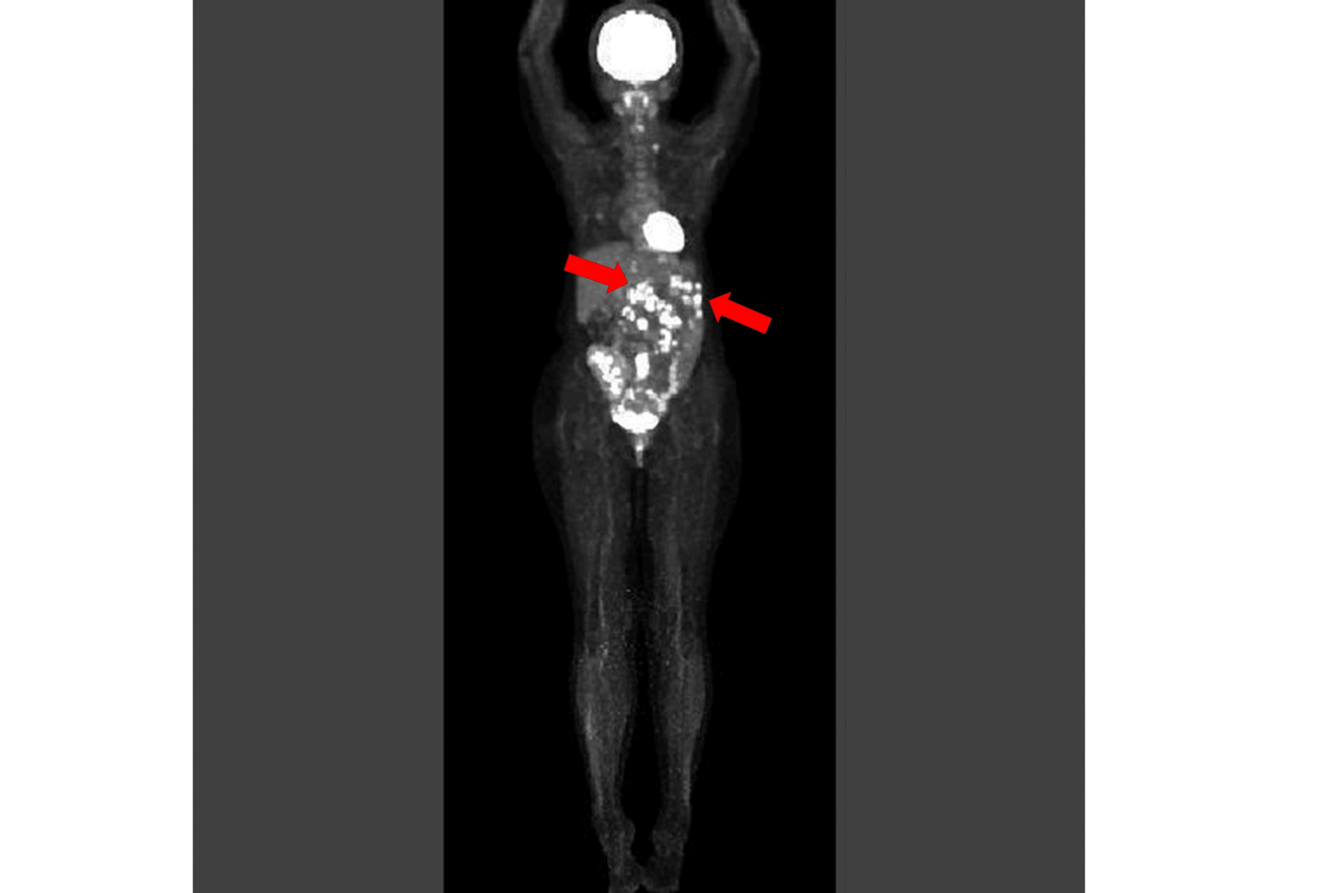
FDG-PET/CT at Initial Diagnosis Fluorodeoxyglucose positron emission tomography/computed tomography (FDG-PET/CT) demonstrates multiple hypermetabolic lymph nodes above and below the diaphragm, consistent with disseminated lymphoproliferative disease. A representative lesion is indicated by an arrow.

Fluorodeoxyglucose positron emission tomography/computed tomography (FDG-PET/CT) demonstrated abnormal uptake in lymph nodes above and below the diaphragm (Figure 2).

**Figure 3:**
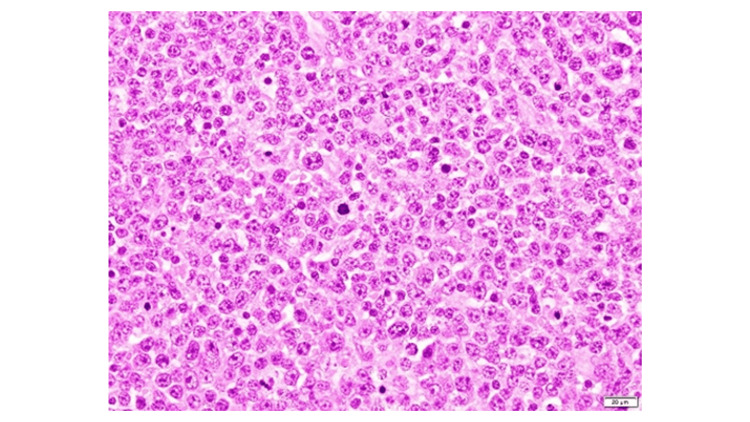
Hematoxylin and Eosin Staining Hematoxylin and eosin staining demonstrates diffuse proliferation of medium- to large-sized atypical lymphoid cells with a high nuclear-to-cytoplasmic ratio, consistent with diffuse large B-cell lymphoma.

**Figure 4:**
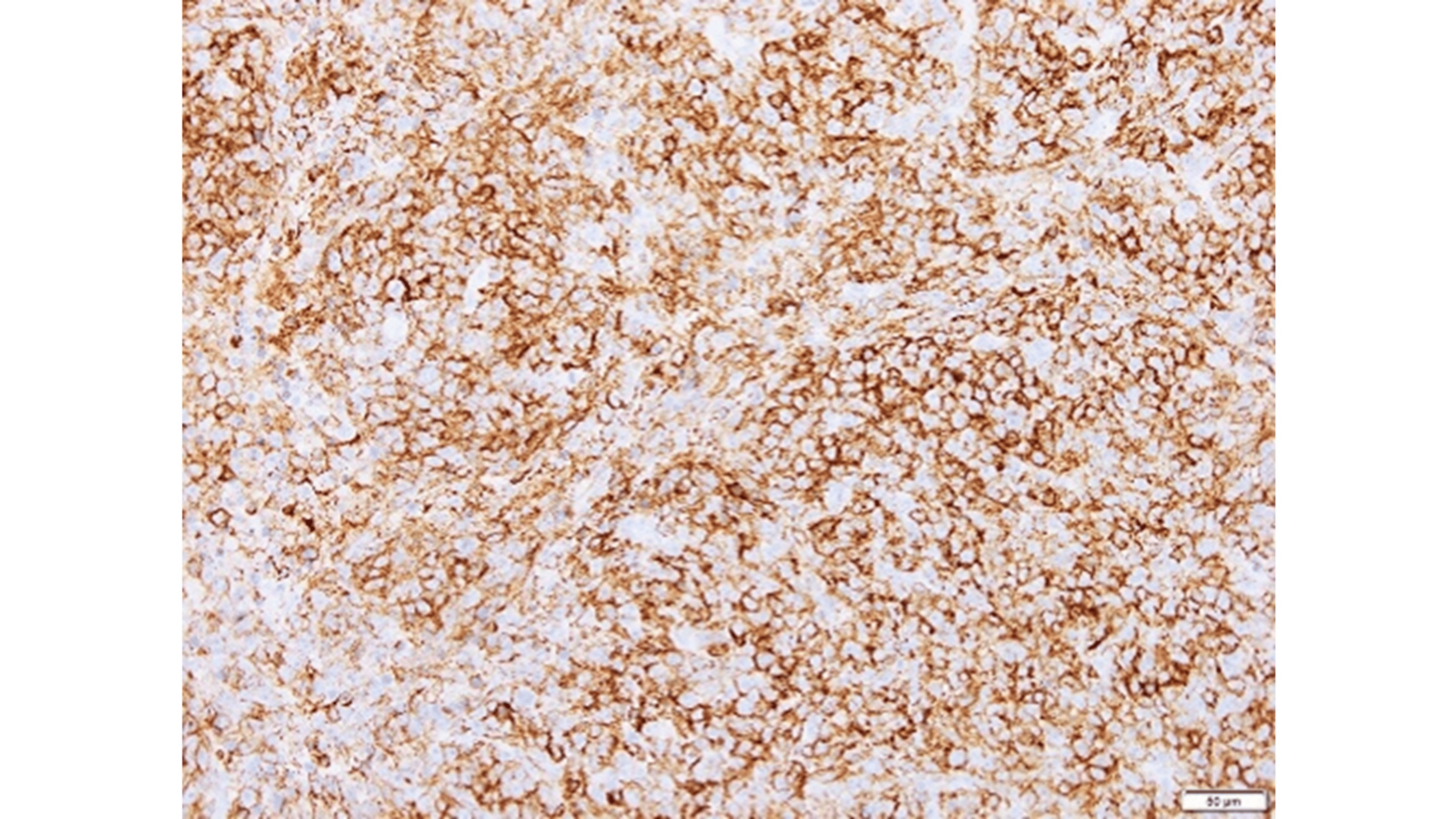
CD20 Immunohistochemical Staining Immunohistochemistry shows strong positivity for CD20 in tumor cells, supporting a diagnosis of B-cell lymphoma.

**Figure 5:**
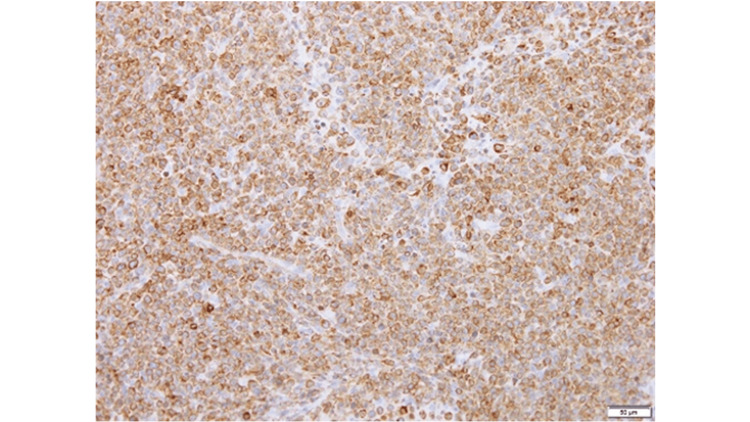
CD79α Immunohistochemical Staining Immunohistochemistry demonstrates positivity for CD79α in neoplastic lymphoid cells, confirming B-cell lineage.

**Figure 6:**
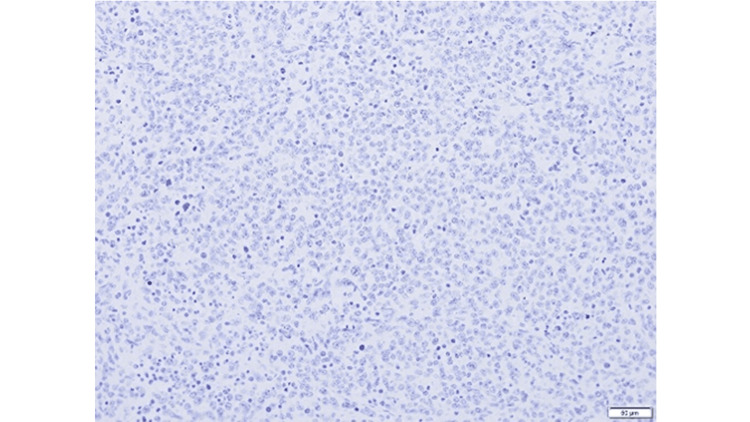
EBER In Situ Hybridization Epstein–Barr virus-encoded small RNA in situ hybridization (EBER-ISH) shows negative staining in tumor cells.

An open biopsy of an intra-abdominal lymph node was performed for definitive diagnosis. Histopathological examination revealed diffuse proliferation of medium- to large-sized atypical lymphoid cells with a high nuclear-to-cytoplasmic ratio on hematoxylin and eosin staining (Figure 3). Immunohistochemistry showed positivity for CD20 and CD79α (Figures 4, 5), while EBV-encoded small RNA in situ hybridization (EBER-ISH) was negative (Figure 6), consistent with DLBCL. Peripheral blood EBV-DNA was mildly elevated at 2.90 log IU/mL.

After diagnosis, the patient was referred to our institution due to relocation. Immunosuppressive therapy was initially maintained without modification. Rituximab monotherapy (375 mg/m²) was administered weekly for eight consecutive weeks.

Interim contrast-enhanced CT after four cycles of rituximab demonstrated a reduction in lymph node size from 20 mm to 8 mm, consistent with partial response. However, at the time of emergency admission following completion of eight cycles, the lymph node had increased to 23 mm, indicating rapid disease progression.

**Figure 7:**
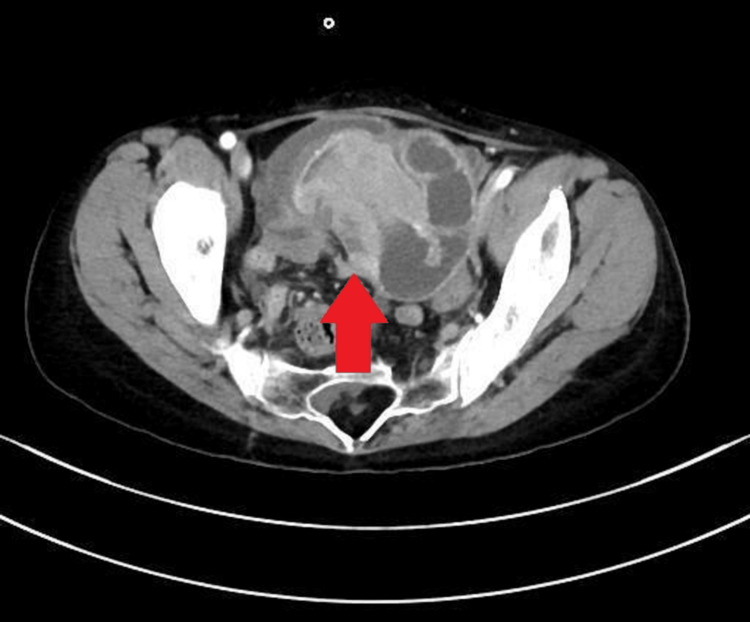
Contrast-Enhanced Computed Tomography (CT) at Relapse CT imaging demonstrates a newly developed small intestinal mass (arrow) causing small bowel obstruction after initial response to rituximab therapy.

Two months after completion of rituximab therapy, the patient developed constipation and abdominal pain, which gradually worsened over approximately 10 days. Initial conservative management, including central venous nutrition, was attempted. However, abdominal radiography revealed findings suggestive of paralytic ileus, and a nasogastric tube was inserted. Contrast-enhanced CT demonstrated small bowel obstruction caused by a newly developed small intestinal mass (Figure 7). Repeat CT performed one week later showed rapid tumor enlargement, and emergency surgery was performed approximately two weeks after admission.

**Figure 8:**
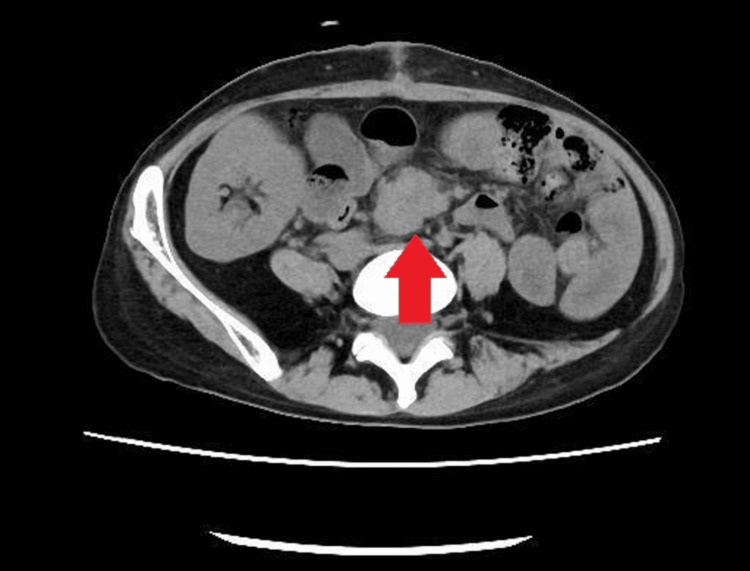
Computed Tomography Showing Disease Progression After Surgical Resection Postoperative CT reveals a newly developed mesenteric mass (arrow) consistent with recurrent diffuse large B-cell lymphoma prior to initiation of systemic chemotherapy.

Surgical resection of the small bowel tumor was performed, and histopathological findings confirmed recurrent DLBCL. Approximately one week after surgery, persistent inability to resume oral intake prompted repeat CT, which demonstrated a newly developed mesenteric mass, indicating disease progression (Figure 8).

During hospitalization, tacrolimus was reduced from 6.4 mg/day to 4 mg/day, targeting a trough level of approximately 3 ng/mL, and was subsequently tapered to 2 mg/day with a target trough level of approximately 2 ng/mL. Azathioprine was continued without dose modification.

**Figure 9:**
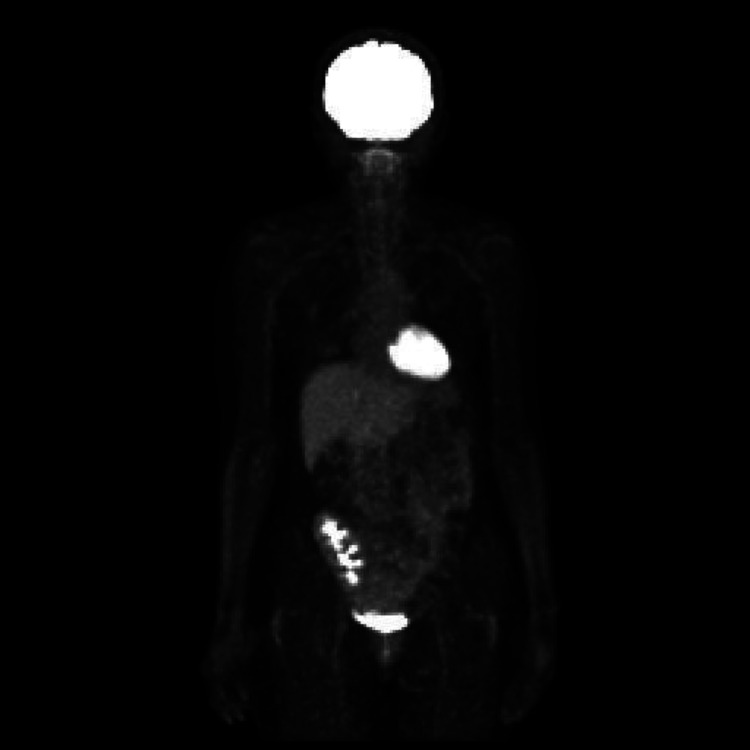
FDG-PET/CT After R-CHOP Therapy Follow-up fluorodeoxyglucose positron emission tomography/computed tomography (FDG-PET/CT) after completion of rituximab + cyclophosphamide, doxorubicin, vincristine, and prednisone (R-CHOP) chemotherapy demonstrates complete metabolic remission with no abnormal FDG uptake.

Two cycles of cyclophosphamide, doxorubicin, vincristine, and prednisone (CHOP) therapy were initiated, followed by four cycles of rituximab + CHOP (R-CHOP). Post-treatment FDG-PET/CT demonstrated disappearance of abnormal FDG uptake, confirming complete metabolic remission (Figure 9).

Following completion of chemotherapy, the patient has been monitored with CT every two to three months and has remained disease-free with stable graft function for six months.

## Discussion

PTLD is one of the most significant malignancies following solid organ transplantation, and its clinical spectrum has evolved with advances in immunosuppressive therapy. The incidence of PTLD after kidney transplantation is approximately 1-3%, whereas higher rates have been reported after small bowel (10-20%), lung (4-10%), liver, and heart transplantation (2-6%) [1]. Although relatively uncommon in kidney transplant recipients, PTLD remains clinically important given the large number of long-term survivors. While the risk is highest within the first year after transplantation, prolonged graft survival has led to increasing recognition of late-onset and very late-onset PTLD, underscoring the importance of long-term surveillance.

PTLD is broadly categorized into early- and late-onset disease, which differ in pathogenesis and biological characteristics. Early-onset PTLD typically occurs within the first year and is strongly associated with EBV infection. Under immunosuppression, impaired EBV-specific cytotoxic T-cell and natural killer cell responses permit uncontrolled proliferation of EBV-infected B cells, leading to tumorigenesis [2]. In contrast, late-onset PTLD, particularly cases arising more than 10 years after transplantation, more frequently present as monomorphic PTLD, most commonly DLBCL, and are more often EBV-negative [3]. These observations suggest that very late-onset PTLD may involve mechanisms distinct from EBV-driven lymphoproliferation, including prolonged immunosuppression, chronic antigenic stimulation, and immunosenescence [4].

Genomic studies further support biological differences between EBV-positive and EBV-negative PTLD. Ferreiro et al. demonstrated that EBV-negative post-transplant DLBCL exhibits genomic and transcriptomic profiles resembling de novo DLBCL in immunocompetent individuals, whereas EBV-positive cases show distinct molecular features [5]. EBV-negative cases more frequently harbor genomic alterations commonly observed in sporadic DLBCL [5-7]. These findings reinforce the concept that EBV-negative monomorphic PTLD may represent a biologically distinct entity with behavior similar to conventional DLBCL.

Therapeutic management of PTLD generally begins with reduction of immunosuppression, balancing tumor control against the risk of graft rejection. For CD20-positive disease, rituximab-based strategies have become central. The prospective PTLD-1 trial demonstrated the efficacy of a sequential approach in which rituximab induction was followed by CHOP chemotherapy in non-responders [8]. Subsequent risk-adapted strategies further supported individualized treatment, allowing low-risk patients to continue rituximab monotherapy while escalating to R-CHOP in higher-risk cases [9].

In our patient, EBV-negative monomorphic PTLD developed 19 years after transplantation and initially achieved only partial response after four cycles of rituximab, followed by rapid disease progression despite continued therapy. This clinical course highlights the aggressive behavior of very late-onset EBV-negative PTLD and suggests that rituximab monotherapy may be insufficient in such cases. Given the biological resemblance to de novo DLBCL, early consideration of systemic chemotherapy such as R-CHOP may be warranted, even in the setting of stable long-term graft function. Importantly, our patient achieved complete remission with CHOP followed by R-CHOP while maintaining allograft function, indicating that curative-intent therapy can be feasible with careful monitoring and appropriate adjustment of immunosuppression.

Previous reports have suggested that very late-onset PTLD may exhibit more aggressive clinical behavior, including rapid tumor progression. In this case, disease progression occurred within a short interval despite initial response, underscoring the need for close imaging follow-up, particularly in patients receiving rituximab monotherapy.

Overall, very late-onset PTLD should not automatically be regarded as a classic immunosuppression-driven disease. Clinicians should maintain long-term vigilance for PTLD even decades after transplantation, as late presentations may occur in otherwise stable recipients. In addition, gastrointestinal involvement may present abruptly with symptoms such as bowel obstruction and should prompt immediate diagnostic imaging and multidisciplinary management. Furthermore, EBV-negative monomorphic PTLD may behave similarly to de novo DLBCL in immunocompetent patients, and early escalation from rituximab monotherapy to systemic chemotherapy such as R-CHOP should be considered in selected cases while carefully balancing graft preservation and treatment-related risks.

## Conclusions

Very late-onset EBV-negative monomorphic PTLD may demonstrate aggressive clinical behavior and early relapse despite initial response to rituximab. In such cases, early consideration of systemic chemotherapy such as R-CHOP may be warranted while carefully balancing graft preservation.
